# Gamma radiation-induced grafting of poly(butyl acrylate) onto ethylene vinyl acetate copolymer for improved crude oil flowability

**DOI:** 10.1038/s41598-024-58521-w

**Published:** 2024-04-17

**Authors:** Ahmed Siddiq, Mohamed M. Ghobashy, Abu-bakr A. A. M. El-Adasy, Ashraf M. Ashmawy

**Affiliations:** 1 Department of Chemistry, Faculty of Science, Al-Azhar University, Assiut, 71524 Egypt; 2 Radiation Research of Polymer Chemistry Department, National Center for Radiation Research and Technology (NCRRT), Egyptian Atomic Energy Authority (EAEA), Cairo, Egypt; 3 Department of Chemistry, Faculty of Science, Al-Azhar University, Cairo, 11884 Egypt

**Keywords:** Gamma irradiation, Grafting, Pour point depressant, Rheology, Crude oil, EVA-copolymers, Flowability, Chemical physics, Polymer chemistry, Chemical synthesis

## Abstract

Ethylene vinyl acetate (EVA) copolymers are widely employed as pour point depressants to enhance the flow properties of crude oil. However, EVA copolymers have limitations that necessitate their development. This work investigated the modification of EVA via gamma radiation-induced grafting of butyl acrylate (BuA) monomers and the evaluation of grafted EVA as a pour point depressant for crude oil. The successful grafting of poly(butyl acrylate) p(BuA) onto EVA was verified through grafting parameters, FTIR spectroscopy, and ^1^H NMR spectroscopy. Treating crude oil with 3000 ppm of (EVA)_0kGy,_ (EVA)_50kGy_, and (1EVA:3BuA)_50kGy_ yielded substantial reductions in pour point of 24, 21, and 21 °C, respectively. Also, rheological characterization demonstrated improving evidenced by a viscosity reduction of 76.20%, 67.70%, and 71.94% at 25 °C, and 83.16%, 74.98%, and 81.53% at 12 °C. At low dosages of 1000 ppm, the EVA-g-p(BuA) exhibited superior pour point reductions compared to unmodified EVA, highlighting the benefit of incorporating p(BuA) side chains. The grafted EVA copolymers with p(BuA) side chains showed excellent potential as crude oil flow improvers by promoting more effective adsorption and co-crystallization with paraffin wax molecules.

## Introduction

Crude oil, often referred to as the "blood of industry" or "black gold," is a crucial component of the global economy^1,2^. However, it is essential to understand that crude oil is a complex mixture containing various substances such as waxes, resins, saturates, asphaltenes, naphthenic, dissolved gases, water, and salts^3^. Among these myriad constituents, waxes, composed mainly of n-alkanes with carbon numbers ranging from 16 to 40, have gained significant attention due to their impact on flow assurance. the content of paraffin waxes within crude oils can range considerably, spanning from 5 to 30 wt.%^4^. Under reservoir conditions, these waxes remain in a liquid state due to high temperatures and pressures^5^. However, In cold weather conditions and deep underground pipelines where temperatures can drop significantly, wax accumulation can occur, thickening pipe walls, reducing pump efficiency, and hindering oil extraction^6,7^. To combat wax deposition, several strategies are employed, including mechanical techniques (scrapers and pigs), thermal methods (insulation, electrical heating, hot oil treatment), and chemical interventions (solvents, dispersants, inhibitors)^8–10^. Among them, Chemical inhibitors, specifically viscosity-reducing agents and pour point depressants (PPDs), are favored for their cost-effectiveness, minimal environmental impact, efficacious outcomes, low energy consumption, and the absence of subsequent processing requirements. Ethylene–vinyl acetate (EVA) copolymer presently stand as the predominant chemical agents employed to mitigate paraffin deposition within pipelines^11–15^. They work through mechanisms such as adsorption, co-crystallization, nucleation, and enhancing wax solubility^16–19^. However, EVA copolymer has certain limitations, including reduced co-crystallization effectiveness with higher vinyl acetate (VA) content and decreasing efficiency with increased paraffin wax content and carbon number. As a result, there is a growing research focus on improving the efficacy of EVA copolymer^20–23^.

Radiation processing of polymers is a widely used method with various applications, including structural modification, polymerization, grafting, sterilization, and crosslinking^24,25^. Grafting procedures are notably favored when executed through gamma irradiation-induced polymerization, distinguishing it from alternatives such as radio-frequency plasma or chemical catalysts^26^. The utilization of gamma radiation offers distinct advantages stemming from its superior penetration capabilities compared to the other two methods. Additionally, it facilitates the attainment of elevated product purity and enables chemical synthesis at ambient temperatures. In contrast, plasma radiation's effect is limited to surface-level polymer modification, while chemical agents frequently yield undesirable byproducts characterized by their potential toxicity and associated high costs^27^.

Acrylic acid and its esters derivatives are one of the prominently employed pour point depressants due to their high-performance proficiency to hinder wax deposition^12^. Yongwen et al.^28^, investigated the modification of alcoholized ethylene–vinyl acetate copolymer (EVAL) by grafting n-alkyl acrylates with diverse lengths of alkyl chain. It was reported that alkyl side-chain insertion could increase grafted EVAL's ability to adsorb and co-crystallize with wax molecules, hence enhancing wax solubility and altering the wax crystallization process. Grigoriy et al.^29^ studied the possibility of improving the physical, chemical, and operational properties of EVA copolymer by grafting the hydrophobic monomers on EVA copolymer using a low-energy electron beam (EB). It was reported that all synthesized grafted EVA polymers performed better in all laboratory and field tests than currently used commercial EVA PPD, and the efficiency of wax inhibition for the Kumkol oil blend reached 90%.

The main goal of this work was to modify Ethylene–vinyl acetate copolymer through induced grafting of Butyl acrylate monomer using gamma irradiation as well as, studying the impact of different monomer concentrations and different gamma doses on the grafting parameters. Moreover, the successful grafted EVA-based copolymer was further evaluated as a pour point depressant for Egyptian Qarun crude oil.

## Experimental

### Materials

Acrylic acid and n-butanol were obtained from Sigma Aldrich. Toluene, methyl alcohol, diethyl ether, p-toluene sulfonic acid and Hydroquinone were obtained from Alfa Aesar. Commercial Ethylene Vinyl Acetate (EVA) copolymer with = 28 wt. % of vinyl acetate (VA) content was purchased from ExxonMobil Chemical Company. Lastly, crude oil was obtained from the western desert of Egypt (QN field — Qarun Company), with a physiochemical characteristic listed in Table 1.

**Table 1:** Physiochemical characteristics of crude oil.

Test	Method	Results
Density@15.5 ℃, g/L	ASTM D-1298	0.79
Specific gravity@60/60 ℉	ASTM D-4052	0.79
API gravity@60 ℉	ASTM D-4052	47.08
Viscosity kinematics at 40 ℃ cSt	ASTM D-445	3.33
Pour point (℃)	ASTM D-97	15
Wax content (wt.%)	UOP 46/64	4.67
Asphaltene content (wt.%)	IP 143/57	0.3
Ash content (wt.%)	IP 4/94	0.001
Carbon residue (wt.%)	IP 13/94	0.44
Sulfur content (wt.%)	ASTM D-4294	0.09
Water content (vol%)	IP 74/70	0.01
Flash point (℃)	IP 170	− 19
Gross calorific value (MJ/Kg)	ASTM D-240	46.37

### Preparations

#### Esterification of acrylic acid and n-Butanol

##### Esterification

The procedure involved conducting the reaction within a four-neck reaction flask equipped with a mechanical stirrer, reflux condenser, thermometer, and a Dean-Stark apparatus. In this process, a solution consisting of 80.14 mmol (5.5 ml) of acrylic acid and 80.14 mmol (7.33 ml) of n-butanol was subjected to reflux in a mixture of 50 mL of toluene. This reaction took place in the presence of 2.5 g of p-toluene sulfonic acid serving as the catalyst, alongside a polymerization inhibitor, specifically 0.25 wt.% (1.45 g) hydroquinone relative to the amount of acrylic acid used. The reaction was maintained at its boiling point (130 ℃) until the calculated quantity of water was distilled out azeotropically^30–33^.

##### Purification of the prepared ester

**Figure 1:**
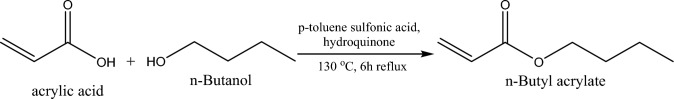
Esterification of acrylic acid and n-Butanol.

The resultant monomer was then distilled under reduced pressure, resulting in the emergence of a white precipitate (Fig. 1). This precipitate underwent multiple purification steps by washing it with a sodium carbonate solution to attain a highly refined product, achieving a yield of 80%^34^.

#### Radiation synthesis of grafted poly butyl acrylate onto EVA

Grafted EVA copolymers were synthesized using a gamma-irradiation induced grafting method. To accomplish this, a sequence was followed. Initially, distinct mass ratios of EVA and BuA, as outlined in Table 2, were dissolved in 20 ml of toluene. Following this, the prepared solution samples underwent irradiation using gamma rays from a ^60^Co Indian irradiation facility gamma ray at a dose rate of 0.866 kGy/h (the establishment of this irradiation facility was overseen by the National Center for Radiation Research and Technology (NCRRT), a division of the Egyptian Atomic Energy Authority (EAEA)), the irradiation procedure was conducted under atmospheric conditions and at room temperature. The polymers resulting from the process were then precipitated using an excess amount of methanol, subjected to filtration, and finally dried under reduced pressure^33,35–37^.

**Table 2:** Different formulations of EVA-grafted polymer.

Sample number	EVA (gm)	Butyl acrylate (gm)	Dose (kGy)	Sample code
1	1	–	0	(EVA)_0kGy_
2	1	–	50	(EVA)_50kGy_
3	1	1	50	(1EVA:1BuA)_50kGy_
4	1	2	50	(1EVA:2BuA)_50kGy_
5	1	3	50	(1EVA:3BuA)_50kGy_

**Figure 2:**
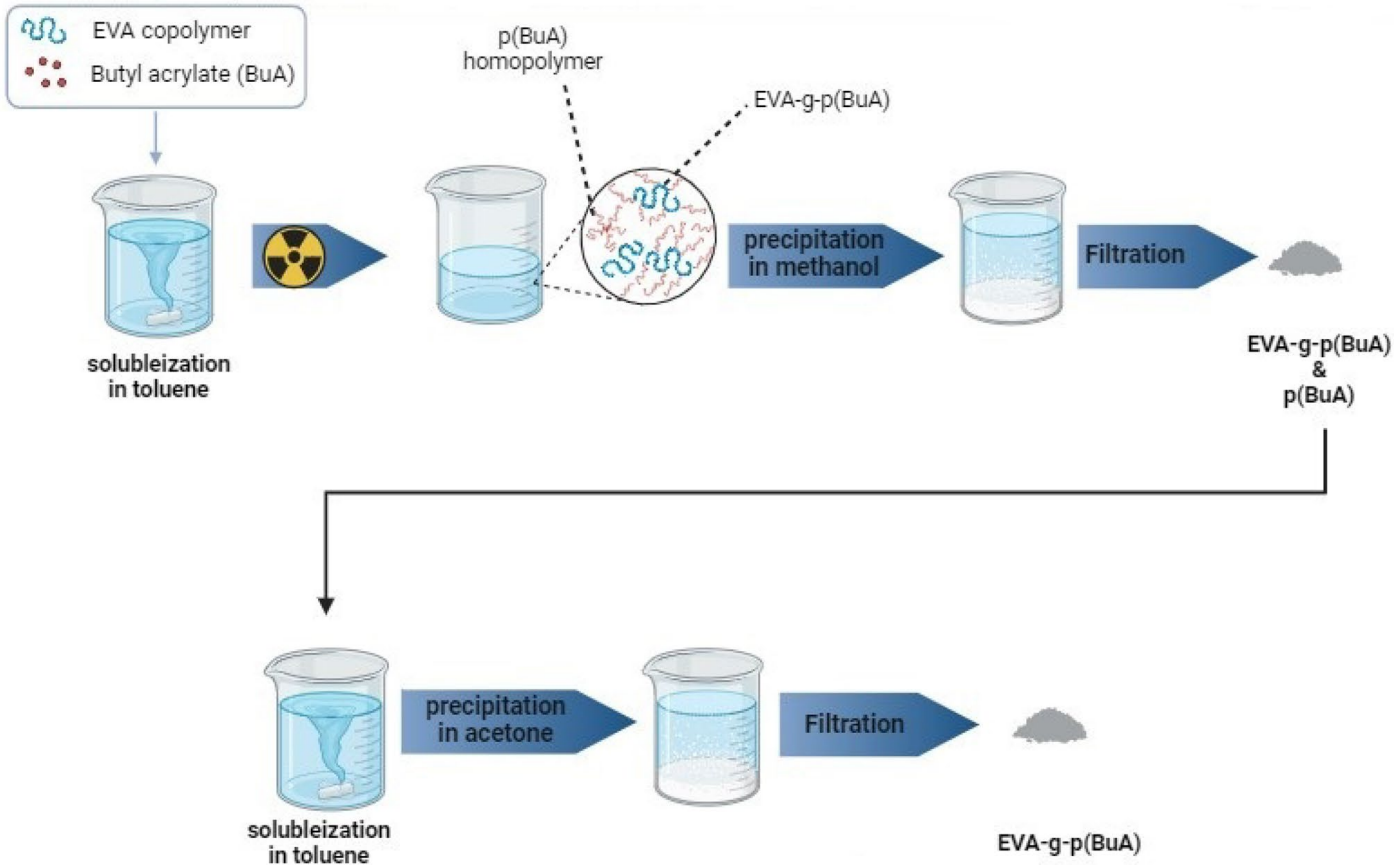
Preparation stages and separation of EVA-g-p(BuA).

During the irradiation of the initial reaction mixture, two processes occur simultaneously: graft copolymerization of BuA monomer onto EVA and Homopolymerization of Butyl acrylate (BuA) monomer resulting in the formation of poly butyl acrylate p(BuA) Homopolymer. To separate the grafted copolymer from the homopolymer, a solubilization-precipitation method was employed. The dried precipitated polymers, which included grafted EVA and the non-grafted p(BuA) Homopolymer, were dissolved in toluene and then precipitated in acetone. This caused the Non-grafted p(BuA) Homopolymer to dissolve while the grafted EVA copolymer precipitated as shown in Fig. 2. The resulting material was filtered and dried under reduced pressure until a constant weight was achieved. Key parameters for evaluating the graft polymerization, such as grafting percentage (G%), grafting efficiency (GE%), and the degree of Non-grafted p(BuA) formation (H_Non-grafted p(BuA)_%), were determined gravimetrically.

### Characterization

#### FTIR spectroscopy

The infrared spectra of the prepared monomer and grafted polymer samples were recorded with a FTIR spectrophotometer (Model: BRUKER ALPHA II) at room temperature (25 ℃), all samples were scanned from 4000 to 400 cm^–1^ with a resolution of 4 cm^–1^.

#### ^1^H-NMR spectroscopy

^1^H-NMR spectra were recorded on a Bruker AVANCE III HD NMR spectrometer (400 MHz) at a frequency of 400 MHz in deuterated chloroform (CDCl_3_) at 25 ℃. Chemical shifts of signals in ^1^H-NMR spectra were determined relative to signals of residual protons CDCl_3_ (7.24 ppm).

#### Determination of grafting parameters

Grafting polymerization parameters for the EVA-based polymers such as; grafting percentage (G %), grafting efficiency (GE %) and Non-grafted p(BuA) Homopolymer ratio (H_Non-grafted p(BuA)_ %) were determined gravimetrically using the below equations^38,39^:

1 $$\mathrm{G }\left(\mathrm{\%}\right)=\left\{\left(\frac{{{\text{W}}}_{{\text{g}}-{\text{EVA}}}-{{\text{W}}}_{{\text{EVA}}}}{{{\text{W}}}_{{\text{EVA}}}}\right)\times 100\right\},$$

2 $$\mathrm{GE }\left(\mathrm{\%}\right)=\left\{\left(\frac{{{\text{W}}}_{{\text{g}}-{\text{EVA}}}-{{\text{W}}}_{{\text{EVA}}}}{{{\text{W}}}_{{\text{m}}}}\right)\times 100\right\},$$

3 $${{\text{H}}}_{{\text{Non}}-\mathrm{grafted p}({\text{BuA}})}\left(\mathrm{\%}\right)=\left\{\left(\frac{{{\text{W}}}_{{{\text{H}}}_{{\text{Non}}-{\text{grafted}}}}}{{{\text{W}}}_{{\text{m}}}}\right)\times 100\right\},$$

where W_g-EVA_, weight of the grafted-EVA copolymer after irradiation; W_EVA_, weight of the EVA copolymer before radiation, W_m_ weight of the BuA monomer charged, W_HNon-grafted p(BuA)_ weight of the Non-grafted p(BuA).

#### Pour point depressant (PPD) test

Pour point measurements were determined using modified ASTM D-97; the oil sample was preheated to 60 ℃ for 1 h to eliminate the thermal history thereof. All oil samples were kept in tightly sealed vessels during heating and a 1-h waiting time to ensure that no light fractions were lost. PPD injection was carried out at 60 ℃ and kept for 30 min at this temperature. During measurements, samples were checked for flow every 3 ℃.

The reduction of the pour point was calculated according to the following equation^40^:

4 $$\mathrm{Reduction\,\, of\,\, pour\,\, point }\left(\mathrm{\Delta PPD}\right)=\left\{{{\text{PPD}}}_{additive}-{{\text{PPD}}}_{pure}\right\},$$

where PPD_pure_ is the pour point of the crude oil without additive (blank) and PPD_additive_ is the pour point of the treated crude oil.

#### Viscosity measurement

The dynamic viscosity of both untreated and treated crude oil samples was measured using the programmable UV-III rheometer by Brookfield. The chosen polymers were selected based on their optimal performance in terms of pour point outcomes. These measurements were conducted at varying concentrations (1000 and 3000 ppm) and across a range of temperatures (12, 25, and 40 ℃).

To quantify the extent of viscosity reduction, we introduce the Degree of Viscosity Reduction (DVR), which can be computed using Eq. (5)^41^:

5 $${\text{DVR}}\left(\mathrm{\%}\right)=\left\{\left(\frac{{\upmu }_{{\text{Non}}-{\text{treated}}}-{\upmu }_{{\text{treated}}}}{{\upmu }_{{\text{Non}}-{\text{treated}}}}\right)\times 100\right\},$$

where µ_Non-treated_ represents the viscosity of the untreated crude oil at a shear rate of 60 S^-1^ in cp, while µ_treated_ represents the corresponding viscosity of the treated crude oil with additives at the same shear rate.

## Results and discussion

### Performed of the prepared esterified acrylic acid

**Figure 3:**
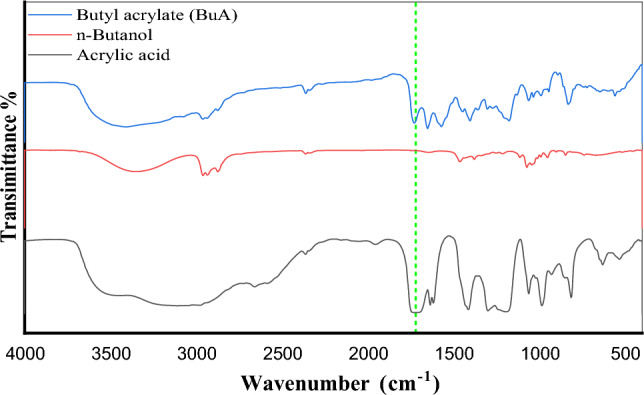
FTIR of the prepared Butyl acrylate monomer.

Butyl acrylate (BuA) is a significant synthetic material appreciated for its desirable properties, including low-temperature flexibility, strong adhesion, hardness, as well as resistance to water and oil. These characteristics make it a valuable raw material for the formulation of paints and coatings, as well as for various other applications such as adhesives^42^. Figure 3 and Table 3 present a comparative examination of the FTIR spectra which agreed with the assignments from previously reported vibrational studies for acrylic acid^43–45^, n-butanol^46,47^, and butyl acrylate^48–50^. The successful esterification of acrylic acid is evidenced by the shifting of the broad band of ν(C=O) from 1707 cm^–1^ to 1727 cm^–1^, also a slight shifting in ν(C=C) was observed from 1635, 1617 cm^–1^ to 1636, 1620 cm^–1^.

**Table 3:** Assignments of the FTIR peaks for synthesized butyl acrylate.

Assignment	Wavenumber (cm^–1^)		
	Acrylic acid	n-Butanol	n-Butyl acrylate (BuA)
ν(O–H)	–	3355	–
ν(COO–)	3109	–	3105
ν(C–H)	2976	2959, 2933, 2874	2961, 2936, 2874
ν(C=O)	1707	–	1727
ν(C=C)	1635, 1617	–	1636, 1620
δ(CH_2_)	–	1463	1464
δ(=CH_2_)	1413	–	1409
ρ(=CH)	1299, 1061	–	1296, 1275, 1064
ν(C–O)	1194	1072	1192
ω(CH=CH_2_)	985	–	985
τ(=CH_2_)	813	–	811

ν stretch, δ scissor, ω wag, τ twist, ρ rock.

### Impact of monomer concentrations and gamma irradiation doses on grafting parameters

The inherent properties of many produced polymers often are not compatible with the desired attributes required for their intended applications in their initial form. Consequently, the necessity arises for modifications to be applied to satisfy specific surface properties. A viable strategy to elevate polymer attributes and introduce novel functionalities involves the utilization of irradiation-induced grafting techniques^51^. This technique can serve to introduce functional groups onto the surface or backbone of the primary polymer, thereby enhancing its overall efficiency across various applications. The grafting yield (measured by increased weight) of the sample after irradiation in the presence of Butyl acrylate monomer is the initial indicator of successful grafting. The different grafting parameters of; grafting percentage (G%), grafting efficiency (GE%) and the ratio of Non-grafted p(BuA) Homopolymer (H_Non-grafted p(BuA)_ %) were further investigated to study its relationship with both Butyl acrylate concentration and gamma radiation dose.

**Figure 4:**
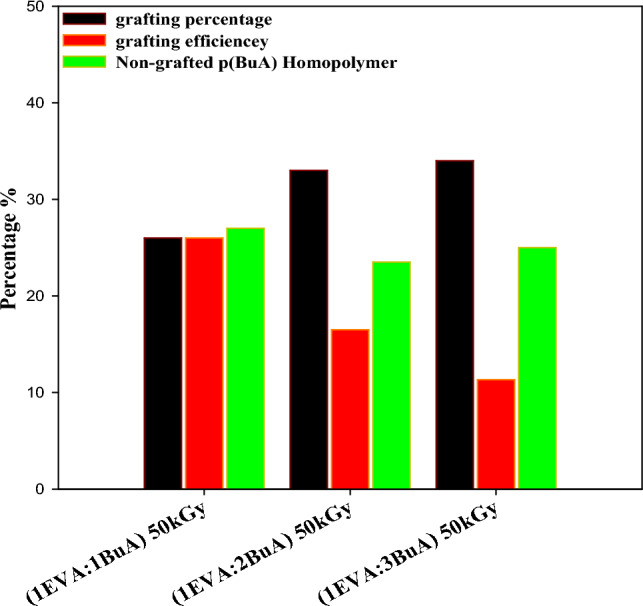
Impact of monomer concentrations of (BuA) on the grafting parameters.

To optimize the monomer concentration, varying quantities of BuA monomer were utilized and the impact of Butyl acrylate (BuA) concentration on grafting parameters is graphically depicted in Fig. 4. It is readily apparent that as the BuA monomer concentration increases, there is a noticeable increment in grafting parameters (i.e. Grafting percentage (G%), Grafting efficiency (GE%), and the degree of Non-grafted p(BuA) Homopolymer formation (H_Non-grafted p(BuA)_%)). This enhancement can be attributed to the higher availability of monomers at elevated concentrations, facilitating their grafting onto the polymer backbone. These findings are consistent with prior research in this field^52^.

**Figure 5:**
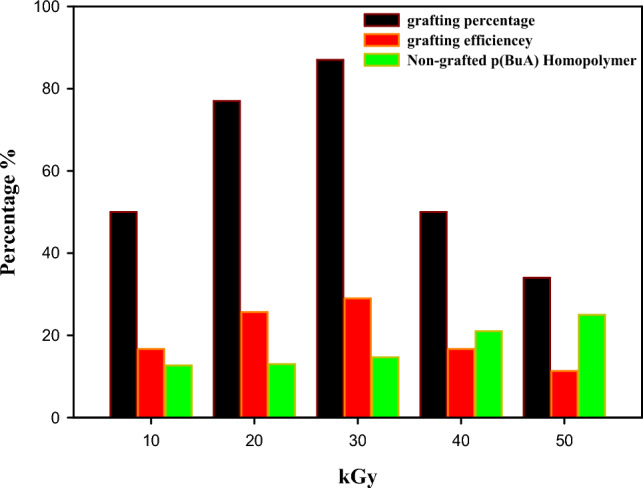
Impact of gamma-irradiation doses on the grafting parameters for 1EVA:3BuA.

Additionally, to investigate the impact of different irradiation doses on grafting parameters, the grafted samples that showed the most effective results as pour point depressants (i.e. (1EVA:3BuA)) were subsequently chosen for an in-depth investigation into the influence of various radiation doses. Figure 5 illustrates the impact of different gamma irradiation doses (ranging from 10 to 50 kGy) on these grafting parameters. It was observed that an increase in gamma dose led to a corresponding increment in grafting parameters (G%, GE%, and H_Non-grafted p(BuA)_%), which can be attributed to the greater provision of high-energy radiation. This enhanced radiation supply facilitates the creation of active sites that encourage the attachment of monomers to the EVA backbone^51,53^. Furthermore, an inverse correlation between the percentage of Non-grafted p(BuA) Homopolymer (H_Non-grafted p(BuA)_%) and the grafting percentage (G%) was observed. This phenomenon can be attributed to the following rationale: When monomer concentration or irradiation dose is higher, Homopolymerization becomes the preferred reaction over grafting polymerization. This can be ascribed to two key factors: (i) As Homopolymer formation increases, the viscosity of the reaction medium rises due to the solubility of Homopolymer in the solvent. This heightened viscosity impedes the diffusion of monomers and the growth of Homopolymeric chains towards active sites, consequently leading to a reduction in grafting percentage^54,55^. (ii) Additionally, the steric hindrance generated by the formed Homopolymeric chains with each other and with diffused monomer further motivates the “grafting to” method rather than “grafting from”, which is characterized by its low grafting percentage as shown in (Fig. S1 (Supplementary Data)).

### Performed analysis of the prepared EVA-g-p(BuA) copolymers

#### FTIR analysis

**Figure 6:**
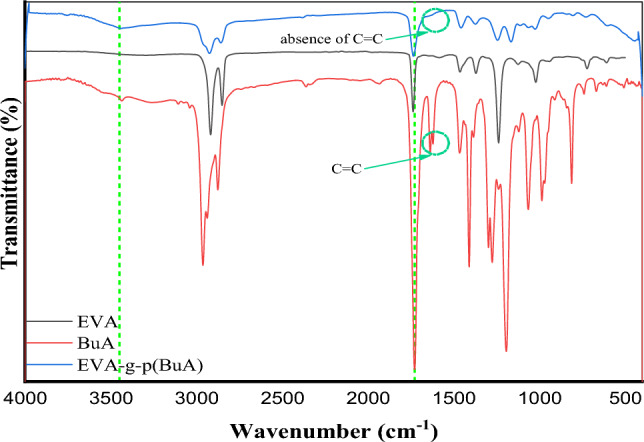
FTIR spectroscopy of EVA, BuA and the grafted EVA-g-p(BuA).

FTIR spectroscopy stands as the predominant analytical approach in the field of polymer research. Its efficacy as a non-destructive method for characterizing polymers and assessing their chemical makeup is widely acknowledged. The pure EVA spectrum in Fig. 6 shown a vibration signal that consistent with vibration signals reported in previous studies^56–59^. Furthermore, as showed in Fig. 6 and elaborated upon in Table 4; The successful grafting of poly butyl acrylate p(BuA) on the EVA backbone was evidenced by the forming of the broad band around 3448 cm^–1^ which could attributed to the Hydrogen bonded between polymer molecules. Additionally, the ν(C=O) was shifted to 1735 cm^–1^, these results come in agreement with a previous works^60,61^.

**Table 4:** Assignments of the FTIR peaks for the prepared EVA-g-p(BuA).

Assignment	Wavenumber (cm^–1^)		
	EVA	n-butyl acrylate (BuA)	EVA-g-p (BuA)
ν(COO–R) & (broad H-bonded)	–	3105	3448
ν(C–H)	2917, 2850	2961, 2936, 2874	2921,2851
ν(C=O)	1736	1727	1735
ν(C=C)	–	1636,1620	–
δ(C–H)	1462 (CH_2_), 1369 (CH_3_)	1464	1460,1374
δ(=CH_2_)	–	1409	–
ρ(=CH)	–	1296,1275,1064	–
ν(C–O)	1237, 1020	1192	1241,1021
ω(CH=CH_2_)	–	985	–
τ(=CH_2_)	–	811	–
ρ(CH_2_)	721	739	721
ω(C=O)	607	–	605

ν stretch, δ scissor, ω wag, τ twist, ρ rock.

#### ^1^H-NMR analysis

**Figure 7:**
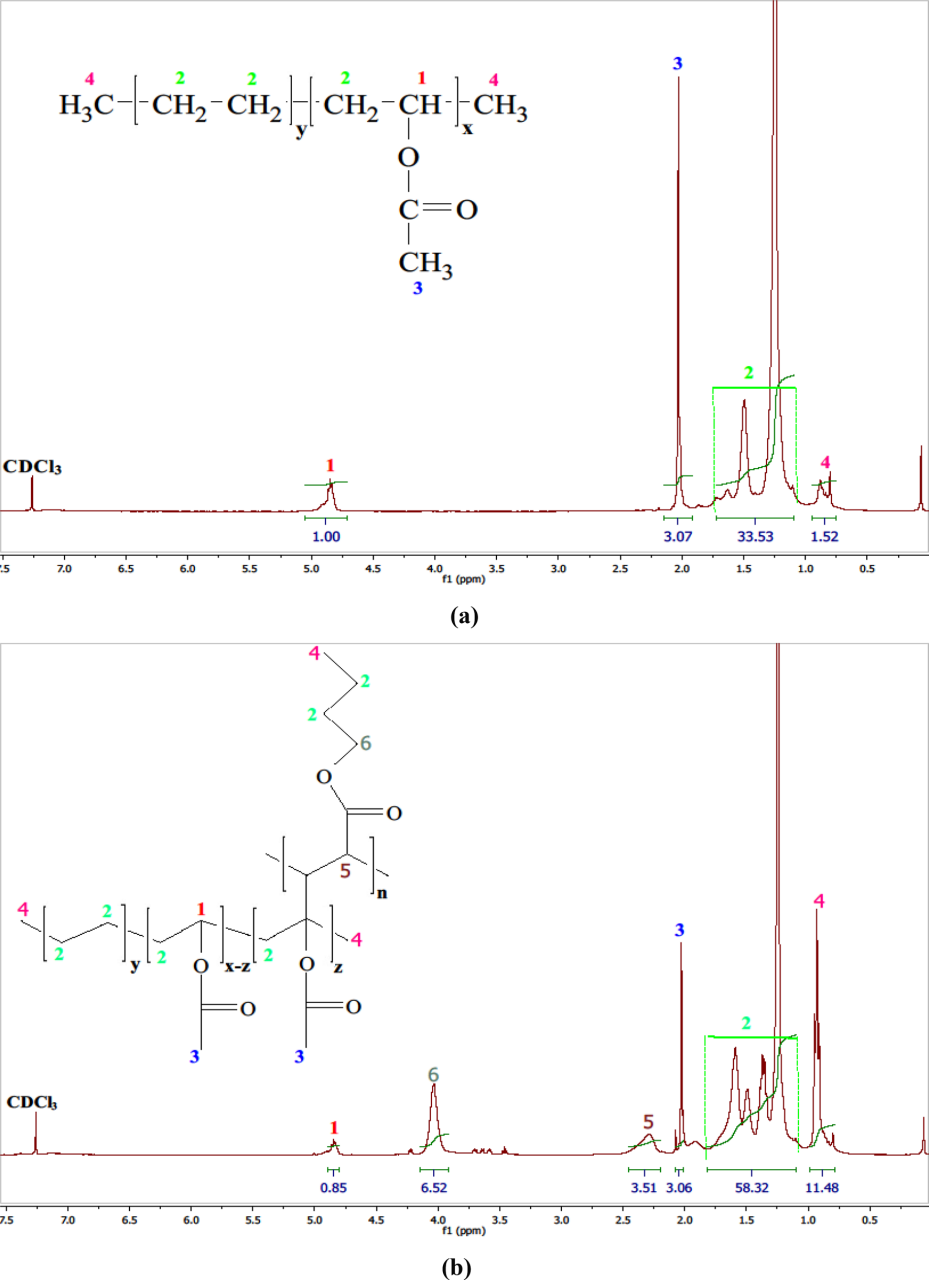
^1^H-NMR spectroscopy of **(a)** EVA, and **(b)** EVA-g-p(BuA).

Nuclear magnetic resonance (NMR) spectroscopy has played a substantial role in enhancing comprehension of the ramifications of radiation on polymeric systems^62^.The significance of NMR as a methodology is underscored by its capacity to enable the precise attribution of signals to individual atoms within both the polymer’s main chain and its side chains^63–65^. The ^1^H-NMR spectra of commercially studied EVA are presented in Fig. 7a. Within the spectrum, the EVA copolymer spectrum exhibits a prominent peak within the 4.8–5.0 ppm range, which is attributed to the methine proton –CH–R– associated with the vinyl acetate moiety (peak 1). Additionally, a peak around δ = 2 ppm is linked to the methyl protons of the vinyl acetate moiety (peak 3). In the δ = 1.1–1.8 ppm range, multiple peaks correspond to the methylene protons found in both the ethylene and vinyl acetate segments (peaks 2). Moreover, the peak representing end-chain methyl protons is observed within the 0.75–0.9 ppm region (peaks 4). These assignments are consistent with previously reported research findings^23,66,67^.

The utilization of nuclear magnetic resonance (^1^H-NMR) spectroscopy for material analysis involves a distinct region known as the compound’s fingerprint region. In our study, we initiated by quantifying the acetate groups within pure EVA copolymer. The vinyl acetate content in pure EVA can be ascertained through peak area integration within the range of 4.8–5 to 1.1–1.8 ppm. Specifically, the peak area denoted as I at 4.8–5.0 ppm corresponds to hydrogen attached to the carbon proximate to the acetate groups, yielding the equation: I_4.8–5 ppm_ = x = (1.00). Within the 1.1 to 1.8 ppm range lies the sum of four methylene hydrogens relative to the ethylene segment in addition to two protons of the acetate segment, resulting in: I_1.1–1.8ppm_ = 4y + 2x = (33.53)^68,69^. Here, the variables y and x represent the fractions of ethylene and vinyl acetate. The molar content of vinyl acetate in the copolymer can be determined through (Eq. 6)^69–71^:

6 $${{\text{VA}}}_{{\text{cont}}.}{\left({\text{mol}}.\mathrm{\%}\right)}_{{\text{NMR}}}=\left\{\left(\frac{{\text{x}}}{{\text{x}}+{\text{y}}}\right)\times 100\right\}.$$

The weight fraction of vinyl acetate (VA_cont._) can be determined using the corresponding VA_cont._(mol %) fraction with the following relationship (Eq. 7)^70,71^:

7 $${{\text{VA}}}_{{\text{cont}}.}{\left({\text{wt}}.\mathrm{\%}\right)}_{{\text{NMR}}}=\left\{\left(\frac{{{\text{M}}}_{\mathrm{VA }}\times {{\text{VA}}}_{{\text{cont}}.}\left({\text{mol}}.\mathrm{\%}\right)}{{({\text{M}}}_{{\text{VA}}}\times {{\text{VA}}}_{{\text{cont}}.}({\text{mol}}.\mathrm{\%}))+{{\text{M}}}_{{\text{E}}}\times {((100-{\text{VA}}}_{{\text{cont}}.}({\text{mol}}.\mathrm{\%}))}\right)\times 100\right\},$$

where M_VA_ and M_E_ are the molecular weight of the vinyl acetate and ethylene (M_VA_ = 86.09 g/mol, M_E_ = 28.05 g/mol), respectively. The result illustrated that the used commercial EVA is (VA(mol%) = 11.26 mol & VA(wt.%) = 28%), which agreed with the actual percent.

In the context of EVA grafting modification, it is reported that the grafting process predominantly occurs on the amorphous vinyl acetate (VA) segments, rather than the crystalline polyethylene (PE) segments^72,73^. Compared to the ^1^H-NMR spectrum of pure EVA some new peaks could be seen in the ^1^H-NMR spectrum of the grafted EVA copolymer, as depicted in Fig. 7b, a novel new peak emerges at approximately 4 ppm, which corresponds to the –O–CH_2_ group of the grafted poly butyl acrylate (peak 6). Additionally, the peak ranging from 2.2 to 2.4 ppm is indicative of the COO–C–H group of the grafted poly butyl acrylate (peak 5). Moreover, an increase in absorbance at the peaks around 0.8 to 1 ppm can be attributed to the heightened presence of end methyl groups within the grafted poly butyl acrylate (peaks 4). These newly identified characteristic peaks provide evidence of the successful integration of the BuA monomer onto the EVA backbone, aligning with findings from several prior investigations^61,74–76^. Finally, upon inspection of the ^1^H-NMR chart of EVA copolymer following grafting polymerization with a modest irradiation dose, a distinct observation emerged. The integration area beneath the curve associated with the methine proton of the vinyl acetate segment exhibited a reduction after grafting. This reduction strongly suggests that grafting of the Homopolymer occurred at precisely this site, with a portion of these protons being substituted by the grafted Homopolymer. Additionally, the application of gamma-radiation induced the ionization of the proton linked to a functional group recognized as possessing electron-withdrawing characteristics.

### Suggested mechanism of grafting polymerization for EVA-copolymer

Graft copolymerization serves as an exceptionally versatile means of functionalization, enabling the incorporation of functional groups into diverse polymer types through the direct polymerization of monomers onto the polymer backbone^77,78^. When grafting polymerization is applied to the EVA copolymer backbone using BuA monomer, two primary approaches are employed, as illustrated in (Fig. S1 (Supplementary Data)):

- “Grafting-to”; this approach involves the attachment of pre-synthesized p(BuA) Homopolymer chains.
- “Grafting-from”; involves the initiation of p(BuA) Homopolymer synthesis directly from the surface, leading to the outward growth of polymer chains^79–81^.

However, the grafting-to method typically results in grafted polymers with lower grafting percentages due to steric hindrance caused by macromolecules already grafted onto the surface. In contrast, the grafting-from synthetic strategy offers the advantage of a broader monomer selection since it’s not restricted by orthogonal monomer side chain functionalities that might interfere with the chain end group used for conjugation, as is the case in the grafting-to approach^82^.

### The impact of EVA-g-p(BuA) on pour point measurement

**Figure 8:**
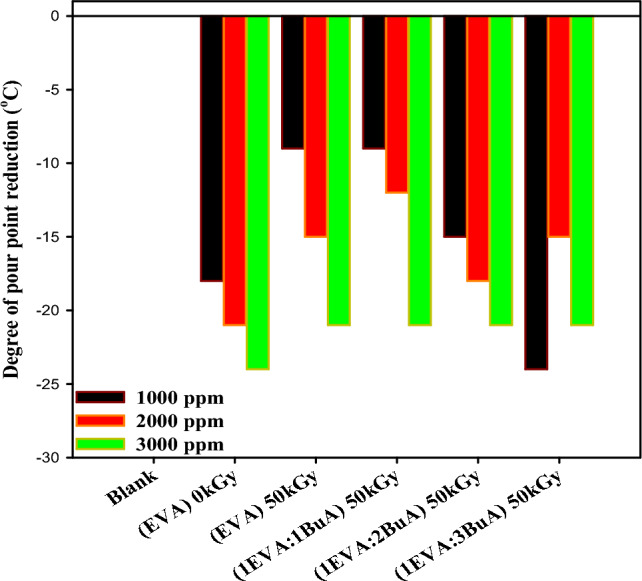
Degree of pour point depressant ΔPPD (℃) with different additives concentrations.

The industry typically employs concentrations of pour point depressants (PPDs) ranging from 50 to 5000 ppm, although there may be some variations^83–87^. The concentration of these additives has a significant influence on pour point depression. Based on the findings presented in Fig. 8, it was observed that increasing the additive concentration from 1000 to 3000 ppm resulted in a noticeable reduction in the pour point temperature^88,89^, and all prepared samples performed well as flow improvers for waxy crude oil. Pure EVA showed the best performance, and the maximum pour point depressant was achieved at a concentration of 3000ppm for (EVA)_0kGy_, (1EVA:3BuA)_50kGy_.

However, each additive has an optimal concentration at which the maximum pour point depression is achieved, but beyond this concentration, the pour point begins to increase. This is primarily attributed to the interaction between additive crystals and wax molecules, which tend to form linkages and promote the adsorption and co-crystallization with the paraffin wax molecule^28,90,91^. Additionally, the grafting of p(BuA) Homopolymer on EVA copolymer results in increased bulkiness and heightened likelihood of intermolecular interaction, thereby diminishing its effectiveness. Consequently, the pour point exhibits a subsequent increase^92–94^. Additionally, as shown in Fig. 8, the most significant pour point depression (ΔPPD = 24 ℃) was achieved with a low concentration (1000 ppm) of (1EVA:3BuA)_50kGy_ which could be attributed to the incorporation of the p(BuA) side chain in the main skeleton of EVA-copolymer. Overall, it can be stated that the grafting of BuA monomer onto the EVA copolymer backbone using gamma radiation {(1EVA:3BuA)_50kGy_} improved the pour point depressant at low concentrations (1000ppm). Also, comparing irradiated and non-irradiated EVA (i.e. (EVA)_0kGy_ & (EVA)_50kGy_) indicates that irradiation had led to crosslinking of EVA copolymer chain thus resulting in decreasing their efficiency. These findings are consistent with those reported by Grigoriy et al.^29^, who found that EVA-based graft copolymers exhibited better PPD performance than commercial pour-point depressants.

A comparative analysis of pour point degrees achieved through gamma-induced grafting of (1EVA:3BuA)_50kGy_, as conducted in this study, reveals notable distinctions from the findings reported by Yongwen et al.^28^. Specifically, our investigation demonstrates that the grafting process resulted in a ΔPPD of 24 ℃ at 1000ppm, surpassing the outcomes of EVAL-g-C_16_ and EVAL-g-C_18_, which yielded ΔPPD values of 11℃ and 15℃ at the same concentration, respectively. Furthermore, the work by Yang et al.^95^ explored the pour point degrees of EVA, EVAL, EVAL-0.5% Carbon nanotubes, and EVAL-1% Carbon nanotubes at 1000ppm, revealing ΔPPD values of 6, 9, 10, and 9, respectively. These findings provide valuable insights into the effectiveness of gamma-induced grafting compared to alternative methods and highlight the superior performance of (1EVA:3BuA)_50kGy_ in achieving significant reductions in pour point temperature.

### The impact of EVA-g-p(BuA) on rheological properties

For most crude oils, when exposed to elevated temperatures, the viscosity remains constant, rendering the chemically intricate crude akin to a straightforward Newtonian fluid. Nonetheless, as temperatures decline, the flow characteristics of crude oil can undergo a transition from uncomplicated Newtonian behavior to intricate flow patterns due to the crystallization of waxes and the colloidal bonding of asphaltenes. Waxes primarily comprise n-alkanes that crystallize, forming interconnected structures of plates and needles. These crystals can ensnare the oil, creating a gel-like arrangement that has the potential to generate substantial deposits within pipelines, leading to heightened pumping pressures that could ultimately obstruct the flow^96–98^.

**Figure 9:**
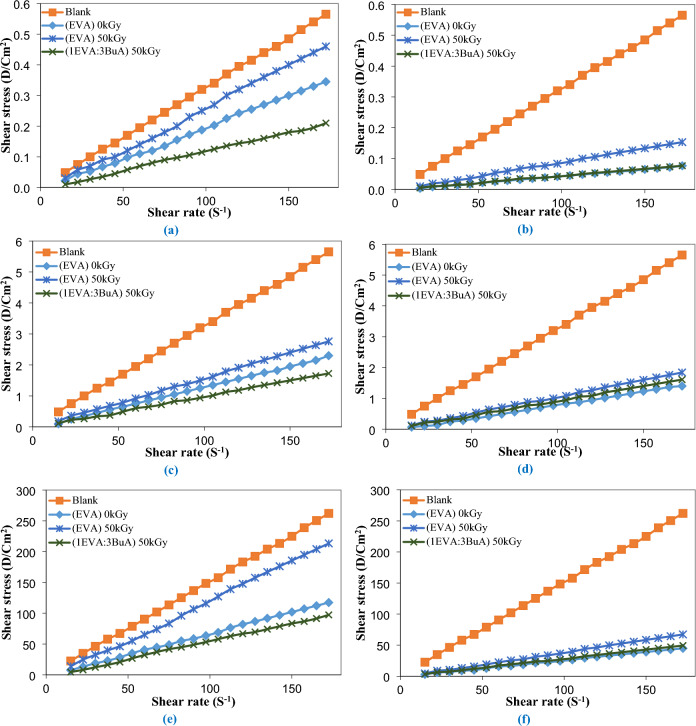
Relationship between shear rate and shear stress for the untreated and treated crude oil using (EVA)_0kGy_, (EVA)_50kGy_, and (1EVA:3BuA)_50kGy_ at temperatures (**a**) 40 ℃, (**c**) 25 ℃, and (**e**) 12 ℃ at 1000 ppm & at temperatures (**b**) 40 ℃, (**d**) 25 ℃, and (**f**) 12 ℃ at 3000 ppm.

**Figure 10:**
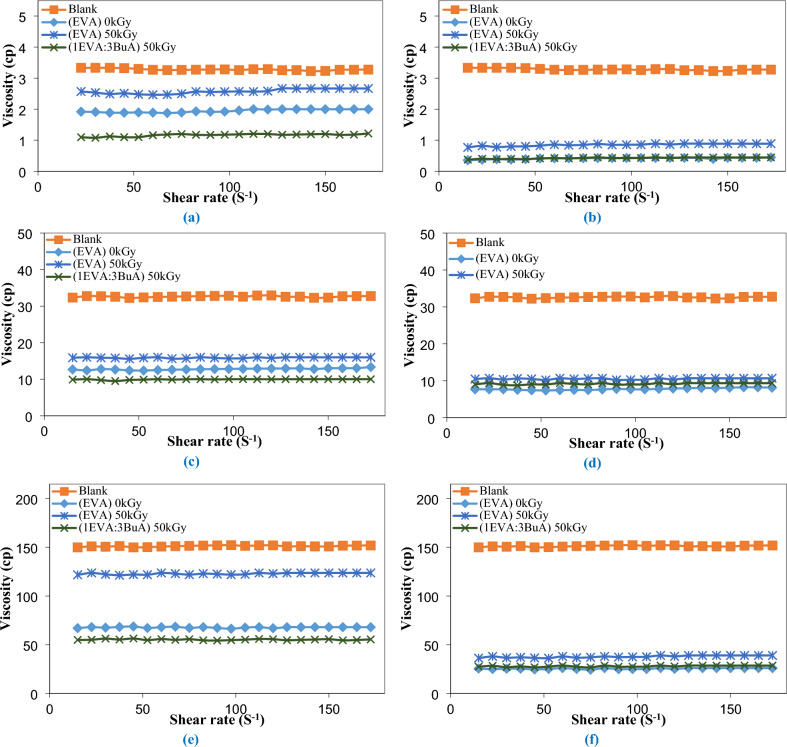
Relationship between shear rate and viscosity for the untreated and treated crude oil using (EVA)_0kGy_, (EVA)_50kGy_, and (1EVA:3BuA)_50kGy_ at temperatures (**a**) 40 ℃, (**c**) 25℃, and (**e**) 12 ℃ at 1000 ppm & at temperatures (**b**) 40 ℃, (**d**) 25 ℃, and (**f**) 12 ℃ at 3000 ppm.

**Figure 11:**
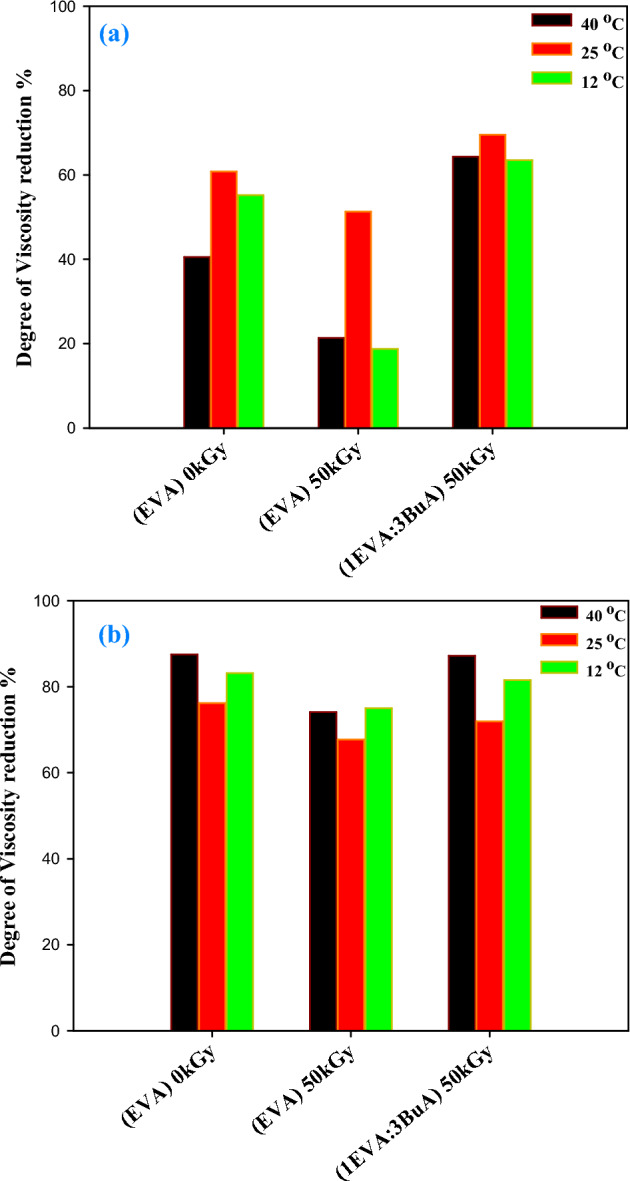
Degree of viscosity reduction (%) treated with (**a**) 1000 and (**b**) 3000 ppm of; (EVA)_0kGy_, (EVA)_50kGy_, and (1EVA:3BuA)_50kGy_ at temperatures; 40, 25 and 12 ℃.

To assess the potential of the prepared EVA-g-p(BuA) copolymers as viscosity improvers for crude oil, the first step involves comprehending the rheological characteristics of untreated crude oil. This entails testing the optimal dosages of both pure and modified EVA-copolymer, which exhibited the most substantial reduction in pour point (specifically, at concentrations of 1000 and 3000 ppm), across a range of temperatures (40, 25, and 12 ℃), that encompassing temperatures both above and below the pour point of the crude oil. The results from the rheological tests are depicted in Figs. 9 and 10, which illustrate the variations in shear stress and viscosity concerning the shear rate for specific samples, namely (EVA)_0kGy_, (EVA)_50kGy_, and (1EVA:3BuA)_50kGy_. Notably, a substantial reduction in the viscosity of crude oil was observed upon treatment with both unaltered and modified EVA copolymers, implying that a lower viscosity at the same temperature corresponds to improved fluidity of crude oil, particularly at lower temperatures^99,100^. Furthermore, as illustrated in Fig. 11, the incorporation of 1000 ppm resulted in a remarkable viscosity reduction of 60.80%, 51.29%, and 69.51% at 25 °C, and 55.20%, 18.74%, and 63.49% at 12 °C. Upon increasing the dosage to 3000 ppm, the viscosity demonstrated even more remarkable decreases, specifically 76.20%, 67.70%, and 71.94% at 25 °C, and 83.16%, 74.98%, and 81.53% at 12 °C, for (EVA)_0kGy_, (EVA)_50kGy_, and (1EVA:3BuA)_50kGy_, respectively.

## Conclusion

The present study investigated the modification of EVA-copolymer via gamma-induced grafting of Butyl acrylate (BuA) which was subsequently evaluated as a flow improver for crude oil. The successful grafting of poly butyl acrylate p(BuA) onto the EVA-copolymer backbone via gamma radiation was confirmed by the characterization of grafting parameters, FTIR and ^1^H-NMR spectroscopy analyses. Additionally, the treatment of crude oil with 3000 ppm of the grafted additives led to considerable reductions in pour point, with depressions of (ΔPPD = 24, 21 and 21 ℃ degree) for (EVA)_0kGy_, (EVA)_50kGy_, and (1EVA:3BuA)_50kGy_. Furthermore, the rheological characteristics of the crude oil showed improvement with the additive’s addition of 1000 ppm evidenced by viscosity reductions of 60.80%, 51.29%, and 69.51% at 25 °C, and 55.20%, 18.74%, and 63.49% at 12 °C. increasing the dosage to 3000 ppm resulted in even greater enhancement in viscosity reductions: 76.20%, 67.70%, and 71.94% at 25 °C, and 83.16%, 74.98%, and 81.53% at 12 °C, for (EVA)_0kGy_, (EVA)_50kGy_, and (1EVA:3BuA)_50kGy_, respectively. Notably, the grafted EVA copolymers with p(BuA) exhibited enhanced performance compared to the native EVA-copolymer even at a low dosage of 1000 ppm indicating that incorporation of p(BuA) as a side chain on EVA-copolymer backbone promoted the adsorption and co-crystallization with the paraffin wax molecule. In addition, irradiation of EVA without introducing monomers led to its crosslinking which showed decreasing in efficiency that was attributed to increasing in its molecular weight.

### Supplementary Information

Supplementary Figure S1.

## Data Availability

The data that support the findings of this study are not publicly available because it is a part of a comprehensive study but available from the corresponding author on reasonable request.
