# Supplementary material for: Gamma radiation-induced grafting of poly(butyl acrylate) onto ethylene vinyl acetate copolymer for improved crude oil flowability
Source: Sci Rep. 2024 Apr 17;14:8863. doi: 10.1038/s41598-024-58521-w (PMC11024112; doi:10.1038/s41598-024-58521-w)
Supplement: Supplementary file 1 — Supplementary Figure S1. [file 41598_2024_58521_MOESM1_ESM.docx]

**Supplementary data**

**Gamma Radiation-Induced Grafting of Poly(butyl acrylate) onto Ethylene Vinyl Acetate Copolymer for Improved Crude Oil Flowability**

**Ahmed Siddiq^1^, Mohamed Mohamady Ghobashy^2^, Abu Bakr A.A.M.El-Adasy^1^Ashraf M. Ashmawy*^3^**

^1^Department of Chemistry, Faculty of Science, Al-Azhar University, 71524, Assiut, Egypt

^2^Radiation Research of Polymer chemistry department, National Center for Radiation Research and Technology (NCRRT), Atomic Energy Authority, P.O.Box.29, Nasr City, Cairo, Egypt

^3^Department of Chemistry, Faculty of Science, Al-Azhar University, 11884, Cairo, Egypt

***Corresponding author:**

Ashraf M. Ashmawy; E-mail: [ashraf_ashmawy2002@azhar.edu.eg](mailto:ashraf_ashmawy2002@azhar.edu.eg)

**Figure S1. Suggested mechanism of grafting polymerization for EVA-Based copolymer:**

Grafting involves the covalent bonding of a monomer or a branched copolymer onto the primary polymer matrix, with the side chain components exhibiting distinct structural characteristics compared to the main polymer chain. Based on the obtained data from the studied gravimetrically grafting parameters, FTIR and ^1^H-NMR spectroscopic results, the gamma induced-grafting of EVA-copolymer could be explained as shown below in **Figure S1**.

## **Figure S1**. Suggested mechanism for grafting polymerization of BuA on EVA backbone.
